# Sources of and Solutions to Toxic Metal and Metalloid Contamination in Small Rural Drinking Water Systems: A Rapid Review

**DOI:** 10.3390/ijerph17197076

**Published:** 2020-09-27

**Authors:** J. Wren Tracy, Amy Guo, Kaida Liang, Jamie Bartram, Michael Fisher

**Affiliations:** 1 The Water Institute, Gillings School of Global Public Health, University of North Carolina at Chapel Hill, Chapel Hill, NC 27599, USA; amy.guo@alumni.unc.edu (A.G.); kliang@email.unc.edu (K.L.); jbartram@email.unc.edu (J.B.); 2 School of Civil Engineering, University of Leeds, Leeds LS2 9JT, UK

**Keywords:** drinking water, water quality, rural water supply, toxic metals, heavy metals, low- and middle-income countries, prevention, correction

## Abstract

Exposure to toxic metals and metalloids (TMs) such as arsenic and lead at levels of concern is associated with lifelong adverse health consequences. As exposure to TMs from paint, leaded gasoline, canned foods, and other consumer products has decreased in recent decades, the relative contribution of drinking water to environmental TM exposure and associated disease burdens has increased. We conducted a rapid review from June to September 2019 to synthesize information on the sources of TM contamination in small rural drinking water systems and solutions to TM contamination from these sources, with an emphasis on actionable evidence applicable to small rural drinking water systems worldwide. We reviewed publications from five databases (ProQuest, PubMed, Web of Science, Embase, and Global Health Library) as well as grey literature from expert groups including WHO, IWA, and others; findings from 61 eligible review publications were synthesized. Identified sources of TMs in included studies were natural occurrence (geogenic), catchment pollution, and corrosion of water distribution system materials. The review found general support for preventive over corrective actions. This review informs a useful planning and management framework for preventing and mitigating TM exposure from drinking water based on water supply characteristics, identified contamination sources, and other context-specific variables.

## 1. Introduction

Exposure to toxic metals and metalloids (TMs) such as arsenic, cadmium, lead, and mercury is detrimental to human health and can have serious, lifelong consequences. Lead exposure alone has an estimated global disease burden of over 100,000 deaths and 10 million disability-adjusted life years per year [1]. Exposure to TMs occurs by dust inhalation; ingestion of contaminated food, water, or soil; and by absorption through the skin [2]. Paint, leaded gasoline, canned food, and other consumer products have historically been major sources of exposure to lead and other TMs. As regulatory actions and process improvements have brought these sources increasingly under control, the relative importance of drinking water as an exposure route has increased [3]. Improvements to analytical instruments have also made it easier to measure and detect TMs in drinking water at concerning levels for health, leading to an increased identification of TM contamination [4].

TMs in drinking water can arise from diverse sources including from natural (geogenic) sources; industrial and mining activities; landfills and waste disposal sites; and water supply and distribution infrastructure itself [5]. For example, one study of arsenic contamination in Bangladesh found that multiple mechanisms likely contribute to the mobilization and release of arsenic into groundwater, including oxidation of mineral deposits, release of arsenic from sediments due to reduction, and release of arsenic from pesticides and fertilizers [6]. Another study in Algeria found that metals in surface water samples likely originated from a mix of industrial and urban discharges as well as agricultural activities [7]. Because of these many sources, control of TMs in drinking water is complex, particularly for implementers and operators of small rural drinking water systems, who may have fewer resources and a lesser technical capacity than operators of large piped systems. While there is information on sources of and possible solutions for TM contamination in drinking water, most of this information is highly technical, focuses on large piped water systems, or is embedded in publications addressing additional contaminants, making it difficult for decision-makers to find. To our knowledge, no simple, robust, practitioner-oriented synthesis of evidence on characterizing and controlling TM contamination in small rural drinking water systems exists.

In response, we conducted a rapid review and synthesis of reviews, expert guides, and grey literature to address the following questions: (i) What are the predominant sources of TM contamination in small rural drinking water systems? (ii) What preventive and corrective actions have been proposed to control contamination from these sources? For the purposes of this review small rural drinking water systems are water systems contained within the community, including point sources such as boreholes and community-managed piped systems, as opposed to large piped systems, which are those connected to a municipal supply.

## 2. Materials and Methods

In June 2019, we conducted a rapid review, which was designed to “[present] a summary of the best available evidence in a synthesized and contextualized manner in direct response to a decision-maker’s question” [8]. Rapid reviews synthesize evidence at an accelerated pace compared to full systematic reviews. They provide decision-makers with a sense of direction and strength of evidence, allowing them to take appropriate action. The World Health Organization (WHO) notes that rapid reviews can produce relevant, demand-driven research that engages decision-makers, and may be especially useful for rapid response mechanisms in low-income settings [8]. Rather than generating new evidence per se, the goal of a rapid review is to provide decision-makers with a timely synthesis of the best available evidence to inform timely policy and practice decisions and actions.

The rapid review followed a four-step process:

1. Identify reviews that include relevant information.
2. Identify grey literature that includes or synthesizes relevant information.
3. Screen identified literature for relevance, emphasizing the evidence most relevant to small rural drinking water systems.
4. Synthesize all relevant literature included after steps 1–3.

Five databases were searched for published peer-reviewed reviews about sources of toxic metals and metalloids (TMs) and solutions for TMs in drinking water systems relevant to small rural communities. This search was conducted with input from national governments from three West African countries and an international non-governmental organization implementing small rural drinking water systems in these countries. The databases searched were Global Health Library, ProQuest, PubMed, Web of Science, and Embase. The search terms used are presented in Table 1.

We also searched grey literature repositories and portals for reports, guidelines, standards, codes, etc. related to TMs in drinking water from expert organizations and groups. Organizations and groups included in the search were the World Health Organization (WHO), United Nations International Children’s Emergency Fund (UNICEF), Joint Monitoring Programme (JMP), International Water Association (IWA), the American Water Works Association (AWWA), Rural Water Supply Network (RWSN), International Association of Plumbing and Mechanical Officials (IAPMO), World Bank, Swiss Federal Institute of Aquatic Science and Technology (EAWAG), Loughborough University Water Engineering and Development Centre (WEDC), International Rescue Committee (IRC), and WaterAid.

All retrieved reviews and grey literature were screened at the title and abstract level, then at the full-text level, using the web-based systematic review software package Covidence (Melbourne, Australia). Inclusion and exclusion criteria are described in Table 2. While the search only included items published prior to June 2019 (the time of the search) and excluded case studies, no other exclusions by date, research design, or reporting method were applied. At all stages, each publication was screened by two reviewers (J.W.T. and A.G.). Conflicts were resolved by discussion between the reviewers.

## 3. Results

### 3.1. Search Results

**Figure 1:**
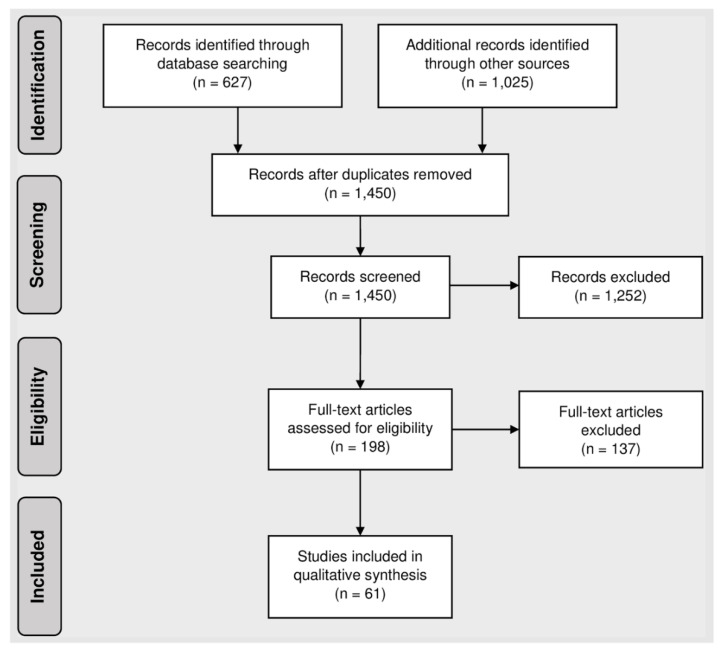
PRISMA diagram for a review of sources of and solutions to toxic metal and metalloid contamination in small rural drinking water systems.

A total of 1450 unique publications were identified through database and grey literature searching (Figure 1). Most publications were excluded at the screening and the full-text review stages because they describe single studies rather than providing a review or synthesis of evidence, or because they were not directly relevant to toxic metals and metalloids (TMs) or drinking water. Information and recommendations from the 61 relevant publications are synthesized in this review.

### 3.2. Sources of Toxic Metals and Metalloids in Drinking Water

The World Health Organization (WHO) states that chemical contamination of drinking water, including TM contamination, can usefully be categorized by source: naturally occurring chemicals; catchment pollution (from industrial activities and human settlements); and chemicals from water treatment and distribution [2,5] (Table 3). Where TM contamination is present in small rural drinking water systems, it is frequently present in the source water and from natural origins as opposed to pollution. However, the probability of TM contamination from each source depends on the TM of interest and regional characteristics including geology, water chemistry, land use, climate, and common water system materials [2,9].

Naturally occurring TMs, such as arsenic, iron, manganese, selenium, and uranium, originate from the earth’s crust and enter water supplies when minerals dissolve [2,10,11,12,13,14]. For example, arsenic and uranium levels in water may be elevated in areas with granite rock formations [12,14,15,16]. Many environmental factors affect the concentration of naturally occurring TMs in catchment waters including surrounding geology, climate, and land use [2]. Natural events also contribute to TM contamination. For example, aluminum, iron, and manganese may be elevated after volcanic ashfall [17] and arsenic may be mobilized from sediment by acid rain [18]. While TM contamination of groundwater is more stable than TM contamination of surface water, TM concentrations can vary widely between individual wells in an area [11]. TM concentrations in shallow wells can also be less stable than those in deep wells, fluctuating based on rainfall [19].

Industrial activities and outputs from human settlements can pollute surface water and groundwater with TMs directly and through atmospheric deposition [19,20]. TM contamination can originate from diverse industrial activities including coal and metal mining; metal smelting and refinement; oil and gas development; electronics manufacturing; pesticide and herbicide manufacturing; and leather and textile processing [20,21,22,23]. Waste from these processes can enter waterways and groundwater through leaking tanks and pipelines; accidental spills; uncontrolled wastewater and stormwater discharges; use of unlined effluent treatment lagoons; and aerial fallout and deposition. While contamination from human settlements predominantly affects urban areas with a greater population density than rural areas, TMs can still enter the catchment from improper sewage and waste disposal as well as fuel leakage in rural areas [2]. Note that while discharges to surface water are more immediately detectable, contamination can take years to decades to travel through soil and enter groundwater supplies [22].

Water treatment and distribution systems can introduce TMs such as copper, lead, iron, and zinc into water through general corrosion. General corrosion, or “partial dissolution of any materials in the plumbing system,” releases dissolved and particulate metals from water system parts, including metal and polyvinyl chloride (PVC) pipes, pipe linings and coatings, solder, joints and fittings, valves and faucets, and even roofing material in rainwater harvesting systems [4,24,25].

Corrosion rates are affected by water chemistry, temperature, contact time, and flow rate. While corrosion chemistry is complex and highly dependent on the metals concerned, lower pH water generally leads to higher rates of corrosion, and the WHO recommends that water be maintained at a pH between 6.5 and 8.5 to minimize corrosion in drinking water [4]. Other factors that generally increase the rate of corrosion include low alkalinity and high temperature as well as higher concentrations of dissolved oxygen and natural organic matter. The impact of other water chemistry parameters including chloride, sulfate, salinity, and hardness on corrosion, while documented, is harder to define given their interactions with each other and with other water chemistry parameters [9,26,27,28,29,30]. Shifts in source water, which result in the introduction of water systems to novel water chemistries, can cause metal release from the water system [25,31]. Stagnant water, because it has had a longer contact time with system materials, is likely to contain higher concentrations of TMs from corrosion. Sudden changes in flow rate, including alternations between stagnation and high flow, are associated with the highest rates of iron release [25,32].

Over time, as pipelines age and protective scales form within the water system, corrosion rates usually decrease, with a few exceptions [25,28,32,33,34]. If the protective scale is dislodged, metals within the scales can be released into the water. Parts within the system that are worn down or damaged are more susceptible to accelerated corrosion [35].

Special types of corrosion may also contribute to TM release. Galvanic corrosion can lead to metal release when electrochemically dissimilar metals are directly connected: for example, contact of leaded or galvanized steel parts and copper parts leads to an increased release of lead into the water system [4,25]. Damaged or worn parts can be susceptible to pitting corrosion, a form of localized corrosion that leads to the creation of small cavities in the material [34,35]. Bacteria can cause biocorrosion through mechanisms such as modifications to water chemistry, deterioration of pipes, and growth and detachment of biofilms that harbor particulate metals [25,32,35,36].

While often overlooked, another contributor to TM contamination is water system installation and construction quality—part of the “water treatment and distribution” category of chemical contamination identified by the WHO. Unsuitable installation and construction practices can inadvertently introduce contamination to the system, increase the corrosion potential of the system, and lead to mechanical problems such as scraping parts [35,37,38]. Particularly problematic is the use of parts including gravel, well screens, pumps, and pipes with a high TM content [39]. Unsuitable post-installation practices, meaning those practices undertaken once the water system has been constructed but prior to its first use by community members, can also cause corrosion and contamination of the system. Insufficient flushing of the system and long stagnation periods after installation can leave construction debris within the system, delay formation of a protective film over piping, and cause pitting corrosion of piping [35].

### 3.3. Solutions to Prevent and Correct Toxic Metals and Metalloids in Drinking Water

Throughout the identified literature, responses for managing TM contamination of drinking water fall into two categories: prevention and correction (Table 4). Preventive actions can be implemented with minimal to no preliminary testing and detection of TM contamination in water systems and frequently involve only a nominal additional cost and effort as compared to corrective actions. For instance, preventing pollution of an initially well-sited and well-constructed water system is less expensive than remediating pollution or having to drill and rebuild a new water system [21]. Preventive actions should therefore be applied across all drinking water systems and situations. Corrective actions, given their inherent complexities and costs, should be implemented once TM contamination has been identified and should be selected and progressively implemented based on contextual knowledge, such as what TMs are present at levels of concern and their known or predicted sources.

#### 3.3.1. Prevention: Best Practices for Siting and Planning New Water Sources

Certain water sources are more vulnerable to pollution (e.g., surface waters and shallow wells) than others (e.g., deep wells) [19]. Selection of higher-quality raw water sources for use in drinking water systems requires less effort and cost than the requisite actions to make lower-quality raw water potable with respect to TM contamination [15]. Therefore, where possible, best practices in siting and planning for new water sources, including testing for known or suspected pollutants and geogenic contaminants, should be employed.

When planning for a new water source, it is important to conduct a water quality risk assessment [40]. Where analytical capacity exists, tests for priority TMs in the raw water to be used for drinking water should be conducted as a direct measure of TM contamination [39]. Other less direct information sources should be assessed in tandem to collect a more comprehensive picture of existing or potential TM contamination. These alternative information sources can include stratified geologic maps, indicating the potential for naturally occurring contamination at the depth of interest, or pollution source mapping, indicating high-risk areas for point source and diffuse pollution [2,15,21,39]. This information can be used to determine the potential areas for drinking water abstraction sites that minimize the risk of TM contamination or, at a minimum, can indicate to the implementing organization which TMs may be present in the water supply, allowing for more targeted planning with respect to materials, treatment, and regulation.

#### 3.3.2. Prevention: Preventing and Reducing Catchment Pollution

A complete separation of pollution from drinking water catchments is unrealistic [19]. To prevent or reduce unnecessary contamination, it is necessary to ensure proper treatment and management of effluent and emissions. Treatment options include recovering TMs from wastewater sludge, removing TMs from industrial emissions through the use of filters and scrubbers, and precipitating TMs from solution before disposal or discharge [20,41]. Other options for directly treating wastewater effluent prior to discharge include anaerobic bioreactors and artificial wetlands [21], but these options may prove cost-prohibitive in some settings. Waste minimization programs should be adopted in tandem with effluent and emission treatment programs; these programs seek to reduce the amount of waste produced, thereby limiting the need for, and the cost of, waste treatment [20].

In support of drinking water catchment protection, governments can strengthen mechanisms to monitor and enforce regulations on TM pollution. Examples of direct regulation options include water quality standards; effluent and emission standards; operational and discharge permits/licenses; and land and water use controls such as zoning based on aquifer pollution vulnerability maps [42,43]. Economic or market-based regulation options also exist but are considered inappropriate for toxic contaminants, since polluters may simply pay to continue polluting [42].

Regulation of TM source water pollution is an important mechanism for preventing and managing their occurrence in drinking water. Considerations for establishing or updating regulations on TMs in source water may be based on a variety of factors including toxicity, geographic extent, and expected TM exposure levels through drinking water [2,5,22]. Given the numerous potential sources of pollution present within a catchment and the differing feasibility of controlling or eliminating each as well as potential limitations on the analytical capacity, resources, and enforcement capacity of the regulating entity, it may sometimes be necessary to adjust standards or to institute a system for progressive realization of health-based standards [5,42]. The progressive realization of standards may, depending on the standard design, encourage immediate action to reduce pollution to currently attainable levels, while also encouraging capacity-building initiatives. Such capacity-building initiatives, including an inventory of all major polluting activities in a catchment area, may subsequently allow for more stringent regulation of TM pollution [5].

#### 3.3.3. Prevention: Using Appropriate Parts and Materials

Water system components containing unsafe levels of TMs can be significant contributors to TM contamination of drinking water [2]. To ensure drinking water system parts on the market do not contain unsafe levels of TMs, national guidelines, international guidelines, or other certification standards should be introduced and enforced [38]. For example, in the USA and Canada, parts sold for drinking water systems are required to contain less than 0.25% lead by mass [44]. Supplier compliance with such guidelines can be audited by governments and implementers through materials testing, where a subset of each lot/batch is inspected to verify that applicable standards are being met [37,38]. Parts certified by third party certification bodies, such as NSF International, the International Association of Plumbing and Mechanical Officials, the International Code Council, Intertek, and UL guarantee compliance with guidelines and standards, reducing the need for further auditing, provided compliance with these certifications can be traceably documented and verified.

While guidelines and standards can limit or eliminate the use of parts with unsuitable TM levels in drinking water systems, the use of inappropriate or damaged parts can still introduce weaknesses to systems, allowing for leaking of TMs into drinking water. In areas where source water is known to be aggressive, water system parts with corrosion-resistant materials such as high-quality PVC or stainless steel should be used [37,45]. It is important to ensure the use of high-quality PVC because in low-quality PVC, the use of TMs as heat stabilizers is unregulated [46]. To prevent galvanic corrosion, it is necessary to avoid the connection of electrochemically dissimilar metals [4,25] and of different grades of the same metal (for example mixing grades of stainless steel) [37]. This is accomplished through the use of dielectric unions. Parts should be inspected by implementers and installers thoroughly for damage before use, as damage can lead to pitting corrosion [35,47]. Guidelines and standards for water system components may differ between potable and nonportable applications, and implementers should ensure that only components certified for potable use are included in drinking water systems.

#### 3.3.4. Prevention: Best Practices for Installation, Construction, and Maintenance

Drinking water systems can become contaminated during their installation and construction [4,38]; therefore, careful installation and construction can reduce TM contamination. Fundamentally, construction should only be entrusted to qualified professionals or properly-trained personnel [4,37]. The WHO advises that the “water authority or governing organization must set standards for the level of training attained by the installation personnel” [4]. Enforcement of training for water system installation personnel should be accompanied by other measures such as licensing and codes of practice to further ensure that proper protocols are followed [48].

Since all water systems, regardless of the material used, require periodic maintenance, it is important that proper maintenance procedures are followed [4,9]. As with installation, maintenance should only be carried out by trained personnel, which helps avoid the introduction of parts with unsafe TM levels and the application of modifications that do not meet system specifications [9,37]. To prevent excessive damage to and corrosion of water system parts, maintenance should be conducted on a consistent preventive schedule, instead of only in response to problems [37,49].

#### 3.3.5. Prevention: Conditioning Water

Conditioning of water can reduce corrosion and system-derived TM exposure [4,25,35,36,50]. This can be achieved with methods such as pH modifications and the addition of corrosion inhibitors, the dosages of which must be adjusted based on water chemistry [35]. The International Water Association (IWA) cautions, however, that the process of handling and dosing chemicals is hazardous and may be difficult for non-professionals to manage [9].

Modifications of pH can be accomplished by contacting water with an alkaline material such as calcium carbonate or magnesium oxide. Alternatively, an alkaline solution of sodium carbonate or sodium hydroxide can be injected to adjust the pH.

Common corrosion inhibitors include silicates, orthophosphates, and polyphosphates. Silicates work by forming a protective film on the surface of the pipe and are more effective at higher pH levels [35]. Orthophosphates reduce the tendency of TMs to dissolve in water and reduce the release of iron, soluble copper, and lead, although the effects on zinc are unclear and there is no reduction in the release of particulate copper [25,35,51]. Polyphosphates reduce pitting corrosion, but may increase overall TM release by preventing protective scale layers from forming [35].

Some practitioners suggest shock chlorination as a method to prevent corrosion [37]. This may reduce biocorrosion, but the overall effect of chlorination on corrosion is still unclear and seems to vary based on the use of free chlorine, chloride, chlorite, monochloramine, or chlorine dioxide; the characteristics of bacteria in the water system; and the presence and characteristics of the scale [25].

#### 3.3.6. Correction: Source Substitution/Blending

While some TMs (such as lead) cannot be consumed safely at any level, other TMs (such as antimony) may be acceptable in drinking water at low levels, and others still (such as iron, selenium, and manganese) are in fact essential elements for human health at low levels [30]. Water sources contaminated with these TMs can be substituted or blended with water from a less contaminated source to reduce the TM concentration [2].

When substituting or blending water sources, there is the possibility of risk substitution, where the new source is also contaminated [31]. For example, the widespread switch from surface water and shallow dug wells to deeper tube wells in Bangladesh in the 1970s–1990s resulted in the substitution of increased arsenic contamination for decreased fecal contamination [52]. Likewise, switching from a TM-contaminated water source to a nearby body of surface water might reduce TM exposure but increase the risk of diarrheal disease [43]. Before using any new water sources for substitution or blending, a risk assessment of each source should be conducted to determine if it meets drinking water standards [2].

#### 3.3.7. Correction: Treating Water

Numerous methods for treatment were identified in the literature including physical filtration (loose media filters, cartridge filters), chemical filtration (activated carbon, manganese dioxide, activated alumina, granular ferric oxide/hydroxide, other locally produced options), aeration, ion exchange, coagulation, reverse osmosis, distillation, and magnetic purification [9,16,43,53,54,55,56,57,58,59,60,61,62]. Each treatment method varies with regards to the TMs it treats, complexity of design, maintenance requirements, and costs. While treatment options may exist that reduce concentrations of all TMs of concern, such options may be cost-prohibitive or unsuitable for a particular water system or community [55,56]. It is therefore important to consider all treatment options available for the TM or TMs of concern and make determinations based on the context and the resources available.

While most of the treatments mentioned in the literature have been tested on the laboratory scale or implemented and monitored in larger, professionally managed water utility systems, in many cases the effectiveness and sustainability of treatment technologies in small rural drinking water systems in low- and middle-income countries has not been described [54]. Still, when determining which treatment to employ for TM contamination, any available evidence on the effectiveness of each treatment method must be considered. Effectiveness of treatment can be an inherent quality of the method, since some methods are more effective than others in treating certain TMs, and can be affected by external factors such as water chemistry; proper installation, operation, and maintenance of the treatment system; and whether the treatment system is used consistently and correctly [52,54]. Appropriate training and community engagement programs can bolster proper setup, maintenance, and use [55,56].

When treatment removes TMs from the contaminated water, this removal process often creates concentrated media or waste, which must be disposed of properly [11]. Care must be taken when planning and operating treatment systems to ensure proper waste management. Without proper disposal, the concentrated TMs could serve as a point source of pollution and could contaminate the water source they were removed from.

As a final consideration, treatment methods, especially when poorly designed, maintained, or operated, do not necessarily achieve complete removal of TMs and do not guarantee that concentrations will be reduced below acceptable health guideline values [54,55]. As a result, treatment methods without proper design, maintenance, operation, and ongoing monitoring may provide a false sense of security and should generally be considered only after prevention and other correction measures have been implemented.

#### 3.3.8. Correction: Correcting Existing Pollution

In most cases where an extraction site is contaminated with TMs, it is simpler and less expensive to find an alternative source of water, especially for small rural drinking water systems that lack professional management [9]. Where there are no safer alternative sources of water, where remediation is required by law, or the contamination site is relatively small, it is possible to remediate contaminated soil and water. The remediation process begins with an evaluation of the contaminant extent and migration pattern in the area. Based on the characterization of the situation, TMs can be removed using methods such as chemical treatment, bioremediation, soil washing, and filtration. For example, iron in liquid waste can be subject to oxidizing treatment before being precipitated out of solution using a base such as slaked lime [41]. Remediation is generally considered difficult and costly [16].

Phytoremediation, or remediation using plants to remove, stabilize, or modify contaminants, may be appealing where technically appropriate because of its relatively low cost [9,59]. Contamination can be restricted from spreading with physical controls and chemical treatments [21].

#### 3.3.9. Correction: Replacing, Modifying, or Cleaning Parts

Where TMs leach from parts used in the water system, the problematic parts should be cleaned, modified, or replaced [2,19,28]. Many of the methods used for cleaning and modifying parts were developed for use in non-potable water systems and gas supply lines, and so may need testing or further modification before use with drinking water systems [63].

To prevent buildup or remove TM contaminants adhered to the sides of a pipe, pipes may be cleaned by flushing with water or compressed air, or by using a “pig” or scraper to physically remove debris [31,32]. This can temporarily reduce contaminant levels, but the sudden changes in water flow can also dislodge protective scale or mobilize particulate TMs, temporarily increasing TM concentrations in water [24,55]. Furthermore, such cleaning may not remove all adhered contaminants (especially if a biofilm is present), is not a permanent solution, and has been criticized as resource-intensive [32].

Another option is to line or coat parts with materials such as epoxy, resin, or polytetrafluoroethylene (“Teflon^®^”) to reduce exposed metal surface area over which corrosion can occur [45,49,63]. The effectiveness of this method is inconsistent. In some cases, flow problems have resulted when epoxy has detached from these linings. However, when executed successfully, linings have been shown to reduce lead concentrations in water. Lining parts may also offer an opportunity for major cost savings—one study of gas pipes in Osaka estimated a 40–60% cost savings by lining pipes rather than replacing them [63].

Parts of the water system can be replaced entirely with new parts with more appropriate composition (e.g., replacement of lead service lines with copper lines) [2,19,28,51,64]. This can be expensive, especially when replacing main lines in large piped networks [28]. Partial system replacement rather than full system replacement can be insufficient as TMs can still be leached from the remaining piping. For example, iron rust on galvanized steel pipes can accumulate and store lead leached from other parts in the system. In a system where all lead piping segments have been removed, the iron rust on these pipes can continue to contribute to lead in tap water for years [24]. However, experts recommend replacing parts with appropriate alternatives, as this is the only way to eliminate the source of TMs.

## 4. Discussion

### 4.1. Toxic Metal and Metalloid Management Framework

**Figure 2:**
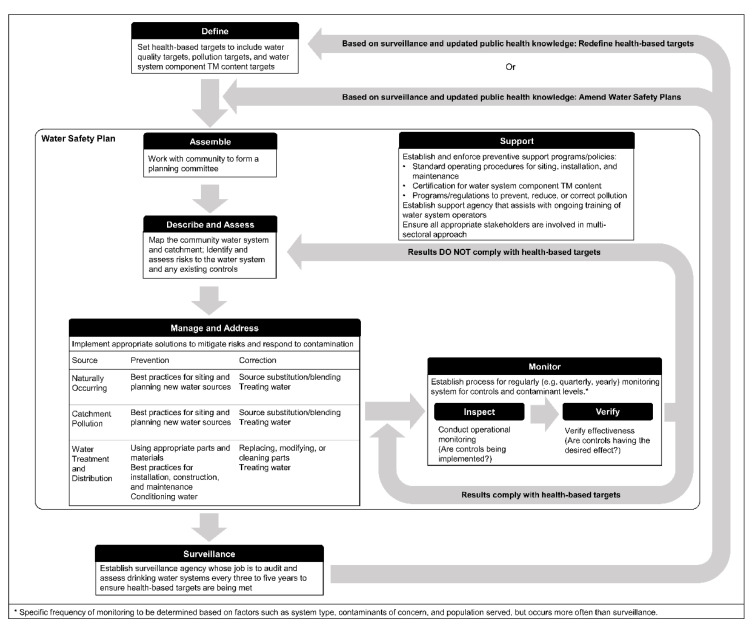
Framework for the management of toxic metal and metalloid contamination.

As the relative contribution of drinking water to toxic metal and metalloid (TM) exposures increases, the need for a structured approach to manage TM contamination of small rural drinking water systems increases as well. Management of TM contamination can be conceptualized within the World Health Organization’s “framework for safe drinking-water” [19,30,40]. The framework encourages a collaborative approach that emphasizes prevention and allows decision-makers to make resource-conscious, incremental improvements that minimize risks to water systems from catchment to distribution to consumer [19]. We produced a framework for the management of TM contamination by incorporating solutions to TM contamination identified in this review into the “framework for safe drinking-water” (Figure 2). This TM management framework can help guide decision-makers through the process of controlling TM contamination. Importantly, this framework narrows the field of solutions, and supports the effective prevention and timely correction of TM contamination in small rural drinking water systems.

As a first step in any TM control program, governments and regulatory agencies will need to review or establish health-based targets for TM contamination. These targets act as guides for progress and provide benchmarks against which outcomes can be assessed [19,30]. Targets should generally be developed and reviewed in consultation with local government authorities, water system operators, engineers, implementers, and community representatives and be incorporated into broader drinking water regulations and standards. Health-based targets should consider implementation and should be “realistic, measurable, based on scientific data and relevant to local conditions (including economic, environmental, social and cultural conditions) and financial, technical and institutional resources” [30]. For effective prevention and management of TM contamination, suitable targets and standards are needed both for TM concentrations in drinking water and TM content in drinking water system components. Suitable targets and regulations on TM pollution are also needed. To be effective, such targets and standards must be supported by monitoring and enforcement mechanisms.

Water safety plans (WSPs) comprise the bulk of activities to be implemented as part of the TM management framework. WSPs encourage communities and system operators, with support from implementers, engineers, and external support agencies, to continuously assess and manage risks to water systems. The WSP approach involves a cycle of system and risk assessments, management of contamination, and monitoring with external support provided by way of programs and policies [19,30,40].

To design a WSP that is suitable for preventing and correcting TM contamination in a specific small rural drinking water system and context, the system operator with community support must conduct a comprehensive system assessment and risk assessment. A proper system assessment and risk assessment should consider all aspects of an existing or planned water system including description and mapping of the catchment, source water, and distribution infrastructure as well as identification of any existing hazards and controls [19,40]. For TM contamination, it is particularly important to know the materials used in the water system and whether the components used have been certified for use in drinking water systems. Risk assessments for TM contamination should not neglect other contaminants in addition to TMs but should instead comprise a broad risk assessment that considers multiple risks to a drinking water system from physical, chemical, microbial, and operational hazards, among others. System assessments and risk assessments should consider all available sources of information including material certifications; water quality tests; community knowledge; expert advice; and complementary information from geological survey departments, mining companies, water supply agencies, nearby utilities, other nearby water systems, wastewater agencies, environmental agencies, major industries, universities, specialist research institutions, departments of trade, industry associations, and material certification entities [2,40]. For small rural drinking water systems, community support is critical to the success of the WSP. Community and local knowledge can often be the most valuable source of information for these water systems when identifying, assessing, and subsequently managing contamination risks [40].

When developing water safety plans, it is often appropriate to include multiple barriers to prevent and manage risks, with source protection and contamination prevention being adopted as the primary barriers in almost all cases. The WHO emphasizes that “the greater the protection of the water source, the less the reliance on treatment” and other corrective actions [30]. Preventive solutions should therefore be applied to all water systems regardless of whether TM contamination has been identified. Indeed, in some cases it may be more cost-effective to implement suitable preventive measures for a potential hazard than to conduct monitoring with sufficient resolution to determine that the hazard in question is absent in all parts of the system throughout the year. While WSPs should be carried out by system operators, effective prevention will require input and assistance from government agencies, especially local government officials; implementers; engineers; and other external support groups and organizations. Support efforts for the prevention of TM contamination, to be overseen by the national government and carried out by local government, include the certification and verification of water system components with respect to TM content; development and verification of standard operating procedures for siting, installation, and maintenance; programs and regulations to prevent, reduce, or correct pollution; monitoring to ensure compliance with and effectiveness of these measures; and training of water system implementers, operators, community leaders, and monitoring teams to assure sustained and effective WSP implementation.

While an effective WSP should emphasize prevention of TM contamination, system operators still must prepare for prompt detection and correction of contamination if it occurs. When correcting TM contamination, system operators will need to use contextual information from risk assessments and ongoing monitoring to consider which solutions to implement and how to adapt them appropriately to the water system and context in question. Efficient use of resources and community engagement is necessary when prioritizing and adapting corrective solutions to TM contamination in small rural drinking water systems, to ensure these solutions can be implemented and sustained over time. Such systems often face challenges that can affect the sustained implementation of solutions including lack of ownership, responsibility, or professional management; lack of implementation and enforcement of regulations; lack of trained staff; and lack of analytical capacity [15]. Given these challenges, system operators should consider this framework as a guide that will help direct them toward appropriate solutions that can be effectively implemented and sustained using the available resources and capacity.

This framework cannot be adequately applied without effective monitoring to ensure proper implementation of solutions and a verification of the effectiveness of these solutions. Any monitoring scheme for controlling TM contamination should include a routine assessment of water systems and testing of water samples, as well as a one-time certification of water system components (at the time of installation when possible, or else at the time of initial WSP development and implementation). Compliance certification of water system components acts as a centralized monitoring function that ensures the suitability, with regards to TM content, of materials in contact with drinking water. Monitoring and regular inspections of the water system itself by the system operator must be conducted as system wear and failures may be indicative of internal component corrosion that could otherwise be missed [35]. Other tools for effective monitoring include checking for water system component certification and operational validation of controls.

Water quality testing can be resource intensive [65], especially in small, rural communities. While there are promising tools in development [31,66], there are currently no low-cost tools for real-time monitoring of TMs in small rural drinking water systems, and only a few validated field tests for a select group of TMs, such as arsenic and iron. As a result, water quality testing should be used by operators to verify that controls are having the desired effect and by national surveillance agencies to characterize the distribution of TM concentrations in drinking water and water system components at the population level. Water quality testing is not currently a feasible tool for high-resolution monitoring to detect short-term fluctuations in TM concentrations within individual small rural drinking water systems.

As the final component of the framework for TM management, surveillance plays an important role in characterizing TM occurrence and exposure through drinking water, in promoting incremental improvements in water quality, and in ensuring that health-based targets are met. Surveillance should be overseen by a national surveillance agency and conducted on a rolling basis, with periodic visits by local officials to every water system at least once every three to five years [19,30]. To better encourage improvements to small rural drinking water systems, the surveillance agency should work through collaboration with water system operators and community representatives. It is important that TM surveillance be incorporated into a broader drinking water system surveillance program that includes other aspects of drinking water quality such as microbial contaminants to ensure efforts are harmonized for all drinking water contaminants. Surveillance agencies can also assist with the training of water system operators and in the dissemination of relevant health information [30].

### 4.2. Limitations of the Review

Because this rapid review limited eligible literature to reviews and guides rather than individual case studies, local innovations and single-study insights were not captured. Furthermore, the effectiveness of identified solutions in small rural drinking water systems was not directly measured or reported in the included evidence. Despite the exclusion of case studies, the review may over-represent evidence from certain geographical regions (North America and South Asia) due to reporting or publishing bias in the overall body of literature. Because the search was limited to English language resources, sources and solutions for TM contamination from non-English speaking regions may not be adequately represented in the included evidence.

### 4.3. Next Steps

Action on TMs requires enabling legislation; institutional commitment and capacity to act; and coordination and transparency among actors and stakeholders. Legislation supports regulatory and enforcement mechanisms to mandate and guide TM contamination prevention and correction activities in drinking water systems. Capacity and commitment are required to design, implement, and monitor these activities. Coordination ensures that prevention and mitigation strategies do not break down at the intersections between implementors, operators, regulators, funders, surveillance agencies, policymakers, and users. Transparency ensures that timely and accurate information flows between these stakeholders and enables the feedback mechanisms that allow WSPs to function freely. Furthermore, transparent surveillance efforts enable consumers to make informed decisions about their drinking water.

Enabling legislation for controlling TM contamination should cover at least four areas: standards, licensing, certification, and consumer protection. Where national drinking water quality standards do not currently specify guidelines for TMs, these should be adopted. Where guidelines exist, these should be reviewed to ensure they are fit for purpose. Policies should also establish requirements and processes for licensing of water system installers and operators as well as certifications of water system components, either through a suitable national government entity or an independent national or international professional body. Consumer protection legislation can support two explicit goals: ensuring the safety of goods on the market intended for the drinking water supply and ensuring compliance of drinking water system implementers with existing laws and regulations. Any implementer that fails to follow the law or uses unsafe products should be subject to sanctions sufficient to discourage repetition which may include the loss of a license to operate, where appropriate.

While suitable enabling legislation is necessary for effective action to control TM contamination, it is likely unnecessary for governments to develop specific legislation dedicated only to TMs. Instead, legislation intended to control TM contamination of drinking water should be incorporated into broader water and materials safety and protection legislation. Where such legislation exists, a review of mechanisms, guidance, targets, enforcement mechanisms, and resource allocation may be useful in ensuring that it effectively enables TM control actions where they are needed.

Legislation should also consider transparency of information on drinking water systems, reflecting the rights of consumers to information on their drinking water and the importance of open data to coordinate efforts across stakeholders. Inclusion of regular reporting to consumers about water quality and water system performance as a requirement for water system licensing may be one approach to make this information generally available. If not already in place, a coordinating entity should be designated to compile, analyze, and report this information from water systems, and integrate the resulting data into national management information systems. The water surveillance agency could, as a part of its mandated duties, be designated to act as the coordinating entity in many settings, ensuring the flow of relevant data and reports to consumers and to local, regional, and national government entities as appropriate.

Future research should investigate robust, low-cost options for monitoring TMs and other associated contaminants and water quality parameters in small rural drinking water systems. Development of simple field test kits or real-time monitoring systems could empower water system operators to conduct more cost-effective and frequent verification of drinking water quality in systems under their supervision. Further research should also appropriately tailor and validate the aforementioned solutions to ensure that they are both financially and culturally sustainable.

## 5. Conclusions

As the relative importance of drinking water as an exposure route for toxic metals and metalloids (TMs) has increased, implementers, operators, and decision-makers at all levels have a growing need for evidence-based solutions and decision-support tools to better prevent and correct TM contamination in small rural drinking water systems. However, relevant information, guidance, and evidence-based solutions are often difficult to locate, vet, and interpret, especially for those outside of research fields. Using a systematic rapid review approach, we synthesized information on sources and recommended solutions for TM contamination in drinking water from relevant reviews and guides. Results led to the development of a TM management framework that adapts the World Health Organization “framework for safe drinking-water” to controlling TM contamination in small rural drinking water systems. The TM management framework emphasizes sector coordination; primary prevention; ongoing monitoring and surveillance; and continuous improvement to organize and optimize actions at the national, local, and system level for cost-effective health protection.

## Figures and Tables

**Table 1:** Search terms used when searching databases for relevant publications.

Keywords	Search Terms
Water	Water, (Drink* OR Potable)
Source Type	tap, tap stand, piped, standpipe, spigot, hand pump, handpump, borehole, tubewell, tube well, private well
Toxic Metals and Metalloids	toxic metal, trace metal, heavy metal, metal, antimony, arsenic, cadmium, chromium, copper, iron, lead, manganese, mercury, nickel, selenium, tin, uranium, zinc
Reviews	review, systematic review, literature review, meta-analysis, meta analysis, guide

**Table 2:** Inclusion and exclusion criteria used to determine eligibility of identified publications for inclusion in the study.

Inclusion Criteria	Discusses or reports on metals or metalloids of interest: antimony, arsenic, cadmium, chromium, copper, iron, lead, manganese, mercury, nickel, selenium, tin, uranium, zinc Discusses source of TM contamination and/or solutions for prevention/correction of contamination Relevant to a drinking water source (or a source likely to be used for drinking such as groundwater or fresh surface water documented as being a source of drinking water for human settlements) Relevant or applicable to small rural drinking water systems Subject to peer review, external review (articles); based on expert consensus/review (book chapters, reports, etc.) Written in English
Exclusion Criteria	Duplicate Case study (descriptive study unable to be generalized because of small scale or unique sociopolitical/geographical context) Focuses exclusively on risk assessment and/or health outcomes rather than sources of and solutions to contamination Is a commentary, editorial, opinion piece, speech, news article, or minutes from a workshop or meeting Has been superseded by a later publication or edition Has been redacted to the point of being unusable Unavailable in English

**Table 3:** Potential sources of contamination for toxic metals and metalloids included in study.

Metal/Metalloid	Potential Sources of Contamination			
	Naturally Occurring	Catchment Pollution		Treatment and Distribution
		Industrial	Human Settlements	
Antimony	X	X		X
Arsenic	X	X		
Cadmium	X	X		X
Chromium	X	X		
Copper		X	X	X
Iron	X	X		X
Lead	X	X		X
Manganese	X	X		
Mercury		X		
Nickel	X	X	X	X
Selenium	X	X		
Tin		X		X
Uranium	X	X		
Zinc	X	X		X

Adapted from the World Health Organization. Guidelines for drinking-water quality: First addendum to the fourth edition, WHO: Geneva, Switzerland, 2017 and Thompson, T.; Fawell, J.; Kunikane, S.; Jackson, D.; Appleyard, S.; Callan, P.; et al. Chemical safety of drinking water: assessing priorities for risk management, WHO: Geneva, Switzerland, 2007.

**Table 4:** Potential solutions for toxic metal and metalloid contamination.

Source	Prevention	Correction
Naturally occurring	Best practices for siting and planning new water sources	Source substitution/blending Treating water
Catchment pollution	Best practices for siting and planning new water sources Preventing and reducing catchment pollution	Correcting existing pollution Source substitution/blending Treating water
Water treatment and distribution	Using appropriate parts and materials Best practices for installation, construction, and maintenance Conditioning water	Replacing, modifying, or cleaning parts Treating water
